# Hepatic candidiasis mimicking lymphoma on ^18^F-FDG PET/CT in a patient with T cell lymphoma

**DOI:** 10.1007/s00259-020-04824-9

**Published:** 2020-04-26

**Authors:** Sandra Schwerz, Marguerite Mueller, Katharina Lindemann-Docter, Alexander Heinzel, Felix M Mottaghy, Mohsen Beheshti

**Affiliations:** 1 Department of Nuclear Medicine, University Hospital, RWTH University, Pauwelsstrasse 30, 52074 Aachen, Germany; 2 Department of Pathology, University Hospital, RWTH University, Aachen, Germany; 3 Department of Radiology and Nuclear Medicine, Maastricht University Medical Center, Maastricht, Netherlands; 4 Department of Nuclear Medicine, Paracelsus Medical University, Salzburg, Austria

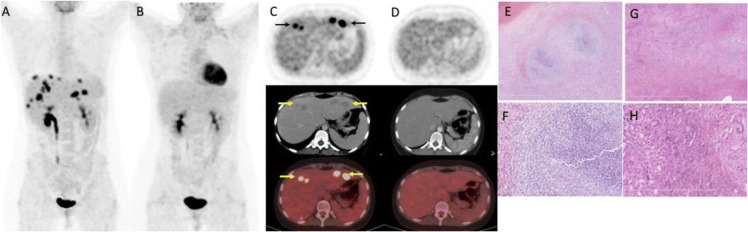



A 19-year-old woman with a history of lymphoblastic T cell lymphoma and mediastinal residual mass after chemotherapy underwent ^18^F-FDG PET/CT for assessment of treatment response. Informed consent was obtained from the participant included in the study. ^18^F-FDG PET/CT showed multiple foci with intensive tracer uptake in both hepatic lobes (A and C—arrows), suggestive of hepatic candidiasis mainly because of the history of previous hepatic candidiasis abscesses (4 months before). However, due to lack of clinical symptoms, intensive ^18^F-FDG avid lesions under antifungal therapy, borderline serum beta-D-glucan (63 pg/mL), and differential diagnosis of liver metastases of lymphoma, further histological verification was performed. The histopathological findings (hematoxylin and eosin stain) revealed focal fibrosis with proliferating bile ducts, foamy cells, and lymphocytes (G and H), correlating with reactive and resorptive processes following the preexisting hepatic abscesses (E and F). Antifungal treatment with posaconazole 300 mg was continued leading to complete remission of the disease assessed by follow-up ^18^F-FDG PET/CT after 3 months (B and D). The residual mediastinal mass showed only faint uptake on both ^18^F-FDG PET/CT studies suggestive of Deauville score 2.

This case presents an unusual pitfall of ^18^F-FDG PET/CT in assessment of liver disease and emphasizes again on the value of ^18^F-FDG PET/CT in the detection of invasive fungal infection, a severe infection in the immunocompromised patients, and also its usefulness in assessing response to treatment [1–4].
